# Glutamatergic Modulators in Schizophrenia: Are We Ready to Move Beyond the Narrow Focus on Dopamine? A Potential Paradigm Shift in Psychopharmacology

**DOI:** 10.31083/AP52026

**Published:** 2026-08-05

**Authors:** Renato de Filippis, Daniel Guinart, Christoph U. Correll, Gaspare Liparota, Taishiro Kishimoto, Pasquale De Fazio

**Affiliations:** ^1^Psychiatry Unit, Department of Health Sciences, University Magna Graecia of Catanzaro, 88100 Catanzaro, Italy; ^2^Institut de Salut Mental, Hospital del Mar, 08003 Barcelona, Spain; ^3^Hospital del Mar Research Institute, CIBERSAM, 08003 Barcelona, Spain; ^4^Department of Psychiatry and Molecular Medicine, Donald and Barbara Zucker School of Medicine at Hofstra/Northwell, Hempstead, NY 11549, USA; ^5^Center for the Promotion of Interdisciplinary Research in Medicine and Life Science (PIRMS), Keio University School of Medicine, 160-8582 Tokyo, Japan; ^6^Northwell, New Hyde Park, New York, NY 11040, USA; ^7^Department of Child and Adolescent Psychiatry, Universitätsmedizin Charité Berlin, 10117 Berlin, Germany; ^8^German Center for Mental Health (DZPG), Partner Site Berlin, 10117 Berlin, Germany; ^9^Einstein Center for Youth Mental Health (ECYM), 13353 Berlin, Germany

**Keywords:** antipsychotic agents, cognition disorders, glutamatergic neurons, receptors, metabotropic glutamate, receptors, N-methyl-D-aspartate

## 1. Introduction

Schizophrenia is a severe and chronic psychiatric disorder characterized by disturbances in perception, thought, behavior and functioning. Clinically, schizophrenia is defined by a heterogeneous constellation of symptoms traditionally grouped into positive symptoms (e.g., delusions, hallucinations, disorganized thought/speech/behavior), negative symptoms (e.g., avolition, anhedonia, social withdrawal), and cognitive impairments involving attention, memory, and executive function. While positive symptoms are often episodic and responsive to antipsychotic treatment, negative and cognitive symptoms tend to be persistent and are the strongest determinants of long-term functional outcome and quality of life [1,2]. Although antipsychotic medications have substantially improved outcomes for many patients since their introduction in the mid-20th century, their therapeutic impact remains uneven across symptom domains. Until recently, antipsychotics, whether first- or second-generation, exert their primary effects through antagonism or partial agonism at dopamine D_2_ receptors, which in many patients effectively reduces positive symptoms but shows limited efficacy for negative and cognitive symptoms [3,4,5]. Over the last two decades, it has become increasingly clear that negative symptoms and cognitive deficits account for the majority of long-term disability in schizophrenia and are only weakly responsive to existing pharmacological treatments [6]. This therapeutic gap has stimulated the search for novel neurobiological targets that extend beyond the narrow focus of dopaminergic neurotransmission. Among these, glutamatergic signaling - and in particular the N-methyl-D-aspartate receptor (NMDAR) hypofunction hypothesis - has emerged as a compelling development within a broader paradigm shift beyond a purely dopamine-centric view of the pathogenesis of schizophrenia [7,8,9].

The pathophysiological models conceptualize schizophrenia as a disorder of dysregulated excitatory-inhibitory balance and disrupted cortical integration, rather than solely as a dopaminergic dysregulation syndrome [10,11]. Within this framework, dopamine dysfunction may represent a downstream consequence of more primary abnormalities in glutamatergic and GABAergic systems.

However, despite strong theoretical and preclinical support, the clinical translation of glutamatergic strategies has been challenging. Multiple compounds targeting glutamatergic pathways have produced mixed or modest effects in clinical trials, and none has yet received regulatory approval [12,13]. This discrepancy between biological plausibility and clinical impact lies at the heart of the current debate: are glutamatergic modulators on the verge of transforming schizophrenia pharmacotherapy, or do their limitations reflect fundamental constraints of this approach?

## 2. Dopamine Versus Glutamate: Conceptual Background

The dopamine hypothesis of schizophrenia, originally formulated in the 1960s, proposed that hyperactivity of dopaminergic transmission - long based on rodent data localized in mesolimbic pathways, now based on human data reconceptualized as being particularly in the associative striatum - underlies psychotic symptoms. This hypothesis, sustained by the psychopathological correlate of aberrant salience, gained strong empirical support from the observation that most of effective antipsychotics mainly target postsynaptic dopaminergic receptors and that dopamine-enhancing agents such as amphetamines can induce or exacerbate psychosis [4,14,15].

In addition, the glutamate hypothesis originated from the observation that NMDAR antagonists such as phencyclidine (PCP) and ketamine can induce a syndrome closely resembling schizophrenia, including negative symptoms and cognitive deficits in addition to psychosis [16,17,18]. This psychotomimetic profile is broader and more phenomenologically similar to schizophrenia than that produced by dopamine agonists, suggesting that glutamatergic dysfunction may occupy a more central position in the disorder’s pathophysiology. The NMDAR hypofunction model proposes that reduced glutamatergic signaling, particularly on inhibitory interneurons, leads to cortical disinhibition and downstream dopaminergic dysregulation [10]. In this framework, dopamine abnormalities are secondary to more fundamental disturbances in glutamatergic neurotransmission. Genetic studies further support this view, as many schizophrenia-associated risk genes converge on synaptic plasticity, glutamate signaling, and NMDAR-related pathways [19,20].

Overall, rather than replacing the dopaminergic model, this glutamatergic perspective complements it, offering a more complete picture in which upstream glutamatergic dysfunction may contribute to and interact with downstream dopaminergic abnormalities.

This conceptual shift may have important therapeutic implications. If schizophrenia is fundamentally or at least significantly a disorder of glutamatergic dysfunction, then directly modulating glutamate transmission might offer benefits across multiple symptom domains, including those inadequately treated by dopamine-based agents. The challenge, however, lies in translating this complex neurobiology into safe and effective pharmacological interventions. Whether glutamatergic modulators can fulfill this promise or whether their role will remain confined to adjunctive and experimental use is the central question that frames this debate.

## 3. Arguments in Favor of Glutamatergic Modulators

### 3.1 Mechanistic Rationale: Addressing Core Pathophysiology

One of the strongest arguments supporting the prioritization of glutamatergic modulators in schizophrenia is their close alignment with contemporary neurobiological models of schizophrenia. Unlike antidopaminergic agents, which primarily modulate downstream neurotransmission, glutamatergic interventions target mechanisms that are increasingly considered central to disease pathophysiology, including synaptic plasticity, cortical connectivity, and excitatory–inhibitory balance [10,11]. The NMDAR hypofunction hypothesis proposes that reduced activity at NMDA receptors, particularly on fast-spiking GABAergic interneurons, leads to disinhibition of pyramidal neurons and abnormal cortical glutamate release. This cascade results in network instability and secondary dopaminergic dysregulation in subcortical regions [21,22]. Such a model provides a unifying explanation for the coexistence of cognitive deficits, negative symptoms, and psychotic phenomena. Importantly, it suggests that dopamine dysfunction may represent a final common pathway rather than the primary cause of schizophrenia.

Glutamatergic modulators attempt to restore physiological NMDAR signaling rather than block downstream effects. These strategies include:

**1. NMDAR co-agonists**, such as glycine and D-serine, which enhance receptor activation at the glycine modulatory site.

**2. Glycine transporter-1 (GlyT1) inhibitors**, such as sarcosine, bitopertin, and iclepertin, which increase synaptic glycine availability.

**3. D-amino acid oxidase (DAAO) inhibitors**, like luvadaxistat, that reduce the metabolism of D-serine.

**4. Metabotropic glutamate receptor (mGluR) modulators**, especially mGluR2/3 agonists and mGluR5 positive allosteric modulators (PAMs), which indirectly regulate glutamate release and NMDAR function [12,13].

This pharmacological diversity reflects the complexity of glutamatergic neurotransmission also related to the fact that glutamate can be neurotoxic and that its levels in the synaptic cleft are therefore tightly regulated by excitatory amino acid transporters (EAAT). However, this diversity also offers multiple potential points of therapeutic intervention. From a theoretical perspective, such approaches are appealing because they aim to correct synaptic dysfunction rather than merely suppress psychotic symptoms.

### 3.2 Targeting Negative Symptoms and Cognition

A central motivation for pursuing glutamatergic treatments is their potential impact on negative symptoms and cognitive impairment, issues that remain partially unresolved by current dopaminergic antipsychotics. NMDAR hypofunction provides a biologically plausible explanation for the pervasive cognitive deficits (including working memory, executive functioning, and social cognition) observed in schizophrenia. Therefore, enhancing NMDAR activity could theoretically improve learning, memory, and executive functioning, thereby addressing a core dimension of the disorder [23].

Recent studies, however, provide preliminary and domain-specific support for glutamatergic strategies. Studies about sodium benzoate, a D-amino acid oxidase inhibitor that indirectly enhances NMDAR function, demonstrated improvements in cognitive performance, particularly in working memory and processing speed [24,25,26]. Similarly, D-serine, an endogenous co-agonist of the glycine site of the NMDAR, has shown some benefits on cognitive measures in several additional small studies [27,28]. However, these findings have been inconsistent across studies, likely due to heterogeneity in baseline glutamatergic tone, disease stage, and dosing strategies. For example, emerging evidence suggests that higher doses of D-serine may be required for central target interaction, raising concerns about dose-dependent nephrotoxicity, which has limited widespread clinical application [28,29].

Indeed, a relevant challenge lies in the nonlinear relationship between NMDAR function and cognition, where both hypo- and hyperactivation may be unfavorable. These findings imply that therapeutic effects may only be observed within a narrow physiological window, reinforcing the desire for biomarker-guided dosing and targeted patient selection. Overall, while glutamatergic modulation remains a promising path for addressing negative and cognitive symptoms in schizophrenia, future progress will depend on more precise phenotyping, improved measurement tools, and mechanism-based experimental designs, compared to indiscriminate targeting of large, only phenotypically defined patient populations.

## 4. Arguments Against the Clinical Prioritization of Glutamatergic Modulators

Despite the compelling theoretical rationale and encouraging preliminary data, several limitations seriously challenge the adoption of glutamatergic modulators in schizophrenia pharmacotherapy. Foremost among these are the mostly negative clinical findings, especially in adequately powered trials. While some trials and meta-analyses report statistically significant benefits, most have failed to demonstrate meaningful advantages over placebo or standard treatment, particularly when rigorous methodological standards are applied [30,31,32]. Another challenge lies in the heterogeneity of schizophrenia itself and by extension the heterogeneity of patients recruited in clinical trials. Schizophrenia encompasses multiple biological subtypes or “endophenotypes”, and glutamatergic dysfunction may be central only in a subset of patients [33,34]. Clinical trials that enroll broad, diagnostically defined populations may therefore dilute treatment effects by including individuals whose symptoms are not primarily driven by glutamatergic abnormalities. Without reliable biomarkers to identify glutamate-responsive subgroups, the therapeutic signal of these agents is likely to remain weak and inconsistent. Furthermore, illness and antidopaminergic treatment effects may attenuate efficacy of glutamatergic modulators, which is why monotherapy trials that do not have to overcome ongoing nonselective dopamine receptor blockade or patients with early-phase schizophrenia may be preferred when assessing the potential value of glutamatergic modulators.

Moreover, safety and tolerability concerns persist. Although most glutamatergic modulators appear relatively safe, certain compounds raise specific issues. High-dose D-serine has been associated with nephrotoxicity in preclinical models, necessitating careful monitoring and limiting its clinical applicability [35].

## 5. Methodological and Translational Challenges

Animal models based on NMDAR antagonism reproduce certain features of schizophrenia, but they cannot fully capture the chronicity, heterogeneity, and developmental complexity of the disorder in humans [36,37]. Consequently, drugs that appear effective in preclinical paradigms may fail to demonstrate comparable benefits in clinical populations.

Another issue concerns outcome measurement. Negative symptoms and cognitive impairment are difficult to assess reliably, and existing rating scales may lack the sensitivity needed to detect subtle pharmacological effects [38]. This issue is further complicated by large placebo effects [39] and by the confounding impact of secondary negative and cognitive symptoms related to depression, medication side effects, or social deprivation [40].

Trial design also plays a critical role. Many studies of glutamatergic agents have involved chronically ill patients with long-standing symptoms and extensive antipsychotic exposure. In such populations, neurobiological changes may be entrenched and less responsive to synaptic modulation. It is possible that glutamatergic interventions would show greater efficacy in early stages of illness, before extensive neural reorganization has occurred [41]. However, ethical and safety concerns complicate the testing of novel compounds in earlier illness stages.

## 6. Future Directions: Toward Precision Psychopharmacology

Rather than abandoning glutamatergic strategies, their future may lie in a more targeted and personalized approach [42]. The integration of clinical biomarkers - such as magnetic resonance spectroscopy measures of glutamate, electrophysiological indices of cortical excitation–inhibition balance, or genetic markers related to NMDAR function - could enable the identification of patient subgroups most likely to benefit from glutamatergic modulation [11].

In this regard, a key priority is the development of patient stratification models based on biological data. For example, glutamate proton magnetic resonance spectroscopy (MRS) studies have consistently demonstrated elevated glutamate or glutamate-glutamine (Glx) levels in subgroups of patients with schizophrenia, particularly in the early stages of the disease, suggesting a hyperglutamatergic state that could predict response to glutamatergic modulators [43,44]. Stratifying patients based on baseline glutamatergic tone (e.g., high versus normal anterior cingulate glutamate) may enrich clinical trials for more likely treatment-responsive subgroups. Electrophysiological biomarkers also offer actionable stratification tools. Deficits in mismatch negativity and auditory steady-state response, which are closely linked to NMDAR-dependent cortical circuitry, have been shown to index glutamatergic dysfunction and predict cognitive and functional outcomes [45,46]. Incorporating such measures as inclusion criteria or enrichment markers may improve signal detection in trials of NMDAR modulators or glycine or d-serine site agonists.

Genetic and molecular stratification represents another promising avenue. Variants in genes related to glutamatergic neurotransmission (such as *GRIN2A*, *GRM3*, and *DAOA*) have been associated with schizophrenia risk and may moderate treatment response [8,47,48]. Polygenic risk scores focused on glutamatergic pathways, or transcriptomic signatures derived from peripheral biomarkers, could further refine patient selection in early-phase trials.

Further, emerging digital and quantitative approaches - such as computerized adaptive testing for rapid and frequent outcome measurement [49], wearable-based activity measures, smartphone-derived behavioral data, or AI-assisted analyses of speech, language, or facial expressivity could improve treatment response assessment [50].

Still, advances in medicinal chemistry, particularly the development of positive allosteric modulators of mGluRs, may overcome some limitations of earlier compounds. By preserving the spatial and temporal dynamics of endogenous glutamate signaling, these agents may offer improved efficacy and safety profiles [51,52]. If successful, they could revitalize the clinical potential of glutamatergic approaches. From a study design perspective, several actions may increase the likelihood of detecting clinically meaningful effects. First, early-phase studies should include biomarkers of target engagement (e.g., changes in glutamate MRS, mismatch negativity amplitude, or functional connectivity) alongside clinical endpoints, as recommended in experimental medicine frameworks [53]. Second, adaptive study designs, such as biomarker-stratified or enrichment designs, may allow for the interim identification of responsive subgroups and dynamic participant assignment [54]. Third, focusing on specific stages of illness may be the key: glutamatergic interventions may be most effective in populations with early or prodromal psychosis, where synaptic dysfunction is likely more plastic and less confounded by chronic dopaminergic dysregulation or antagonist treatments [34,55].

## 7. Conclusions

The debate surrounding glutamatergic modulators in schizophrenia reflects a broader tension between neurobiological innovation and clinical pragmatism. On the one hand, these agents are grounded in a sophisticated and increasingly influential model of schizophrenia as a disorder of synaptic and circuit-level dysfunction. They offer the promise of addressing negative symptoms and cognitive deficits, which remain among the greatest unmet needs in contemporary treatment. On the other hand, their clinical effects have so far been inconsistent, modest at best, and limited primarily to adjunctive use, without sufficient evidence for their approval as primary treatments for schizophrenia, let alone replacing antidopaminergic antipsychotics as the cornerstone of therapy.

Thus, glutamatergic modulators should neither be dismissed as translational failures nor prematurely embraced as paradigm-shifting treatments. Instead, they may represent an additional component of a more nuanced, multi-system approach to schizophrenia pharmacotherapy. Their ultimate value may lie not in supplanting antidopaminergic treatments, but in complementing them within a precision medicine framework that acknowledges and leverages the biological heterogeneity of the disorder.

Hence, the question is not whether schizophrenia treatment should move “beyond dopamine”, but rather how dopaminergic and glutamatergic strategies can be integrated to more effectively address the full spectrum of symptoms and functional impairments. The future of schizophrenia psychopharmacology may therefore depend less on replacing old paradigms than on combining them in a scientifically informed and clinically flexible manner, as is currently seen since the first approval of the muscarinic M1-M4 agonist xanomeline-trospium chloride.
